# Supplementary figures and images for: Two Complete Genomes of Male-Killing Wolbachia Infecting Ostrinia Moth Species Illuminate Their Evolutionary Dynamics and Association with Hosts
Source: Microb Ecol. 2023 Feb 22;86(3):1740–54. doi: 10.1007/s00248-023-02198-7 (PMC10497655; doi:10.1007/s00248-023-02198-7)

Fig. S1

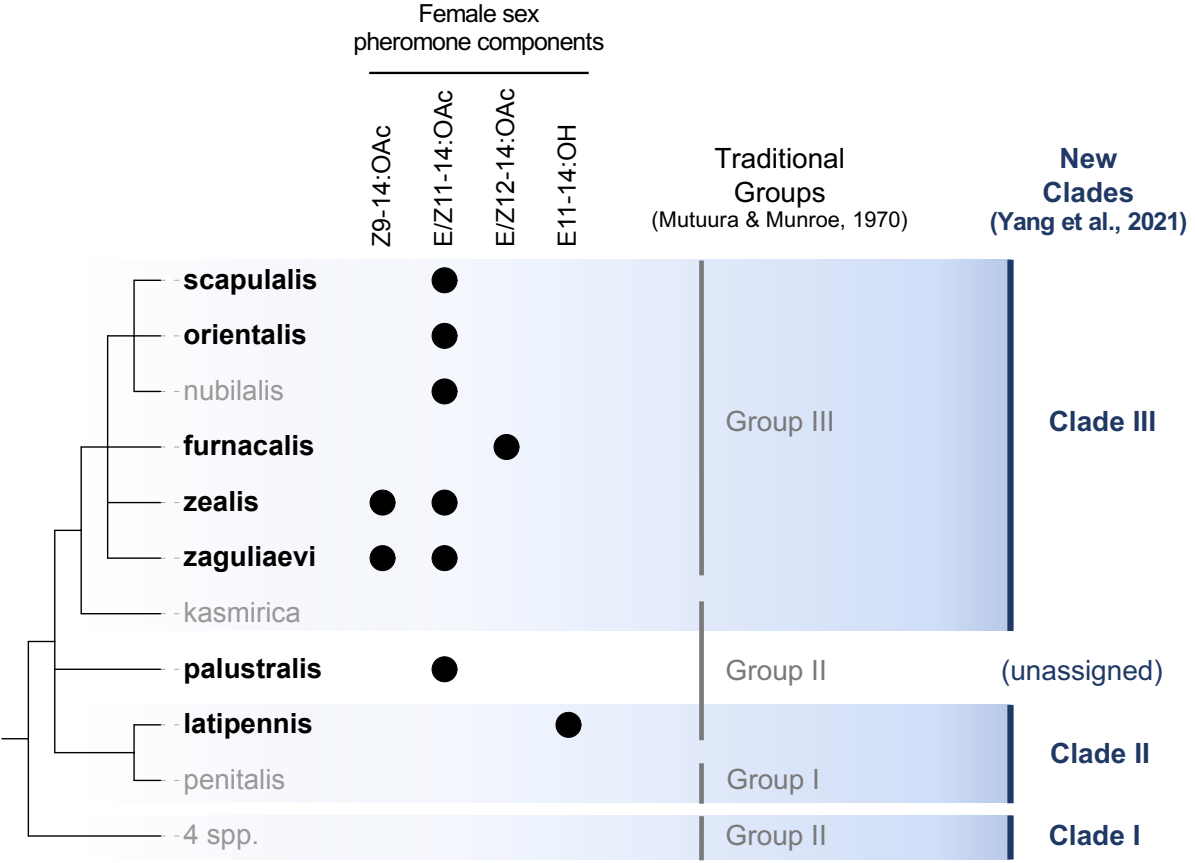

Fig. S2

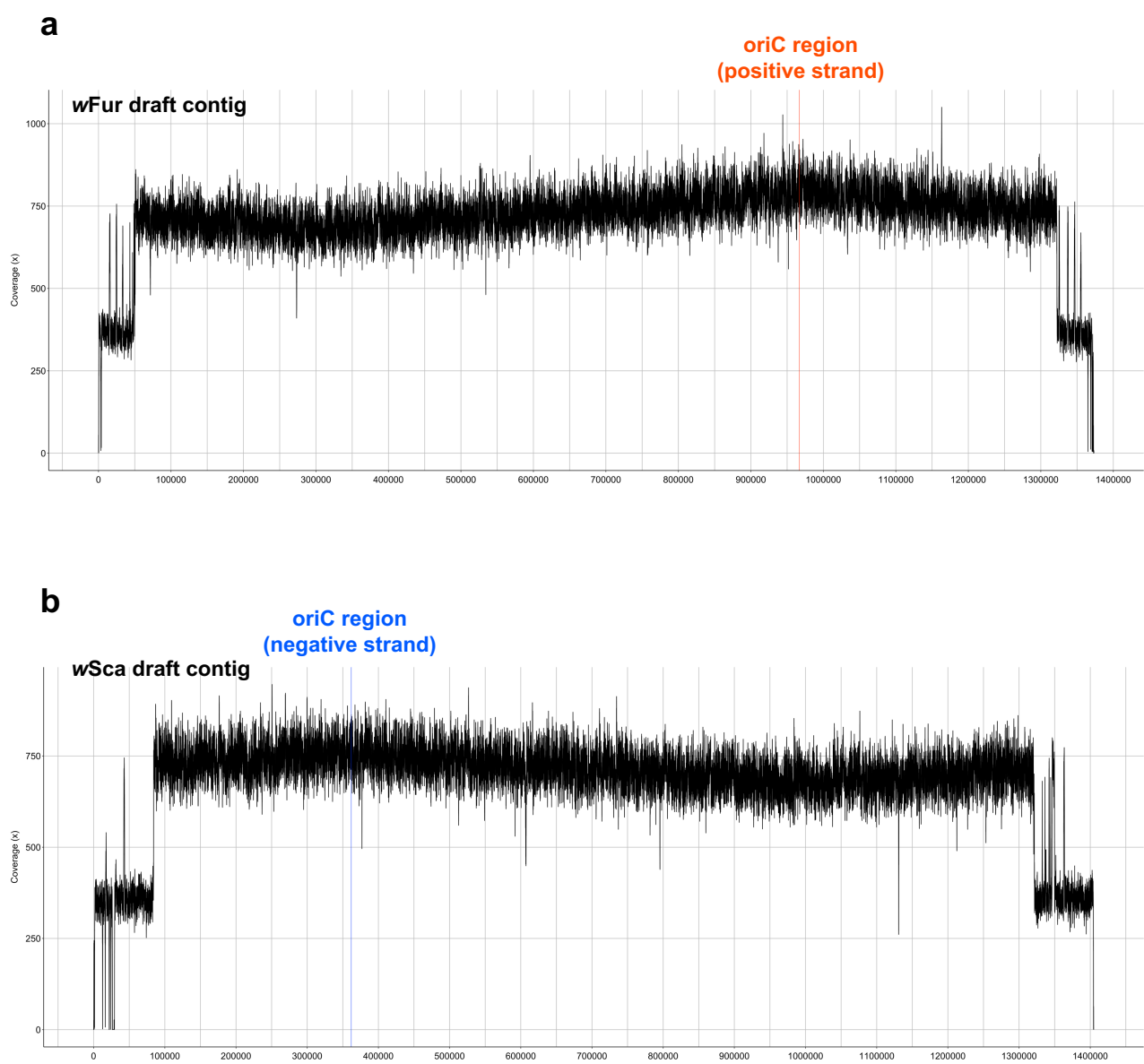

Fig. S3

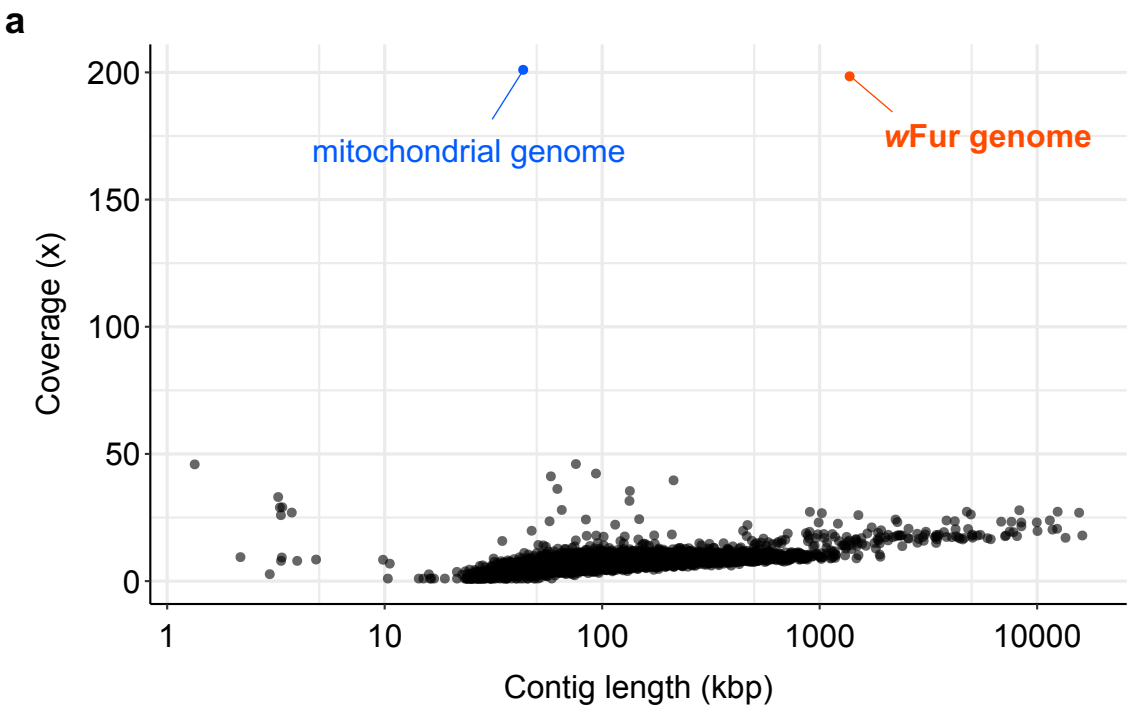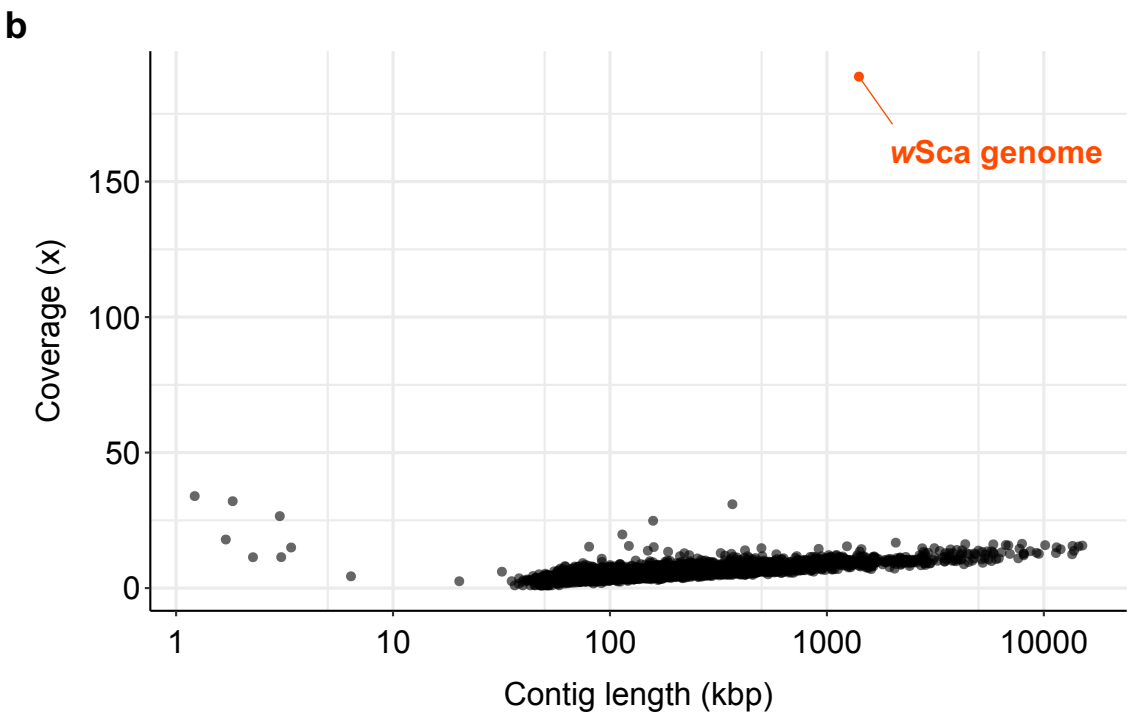

Fig. S4

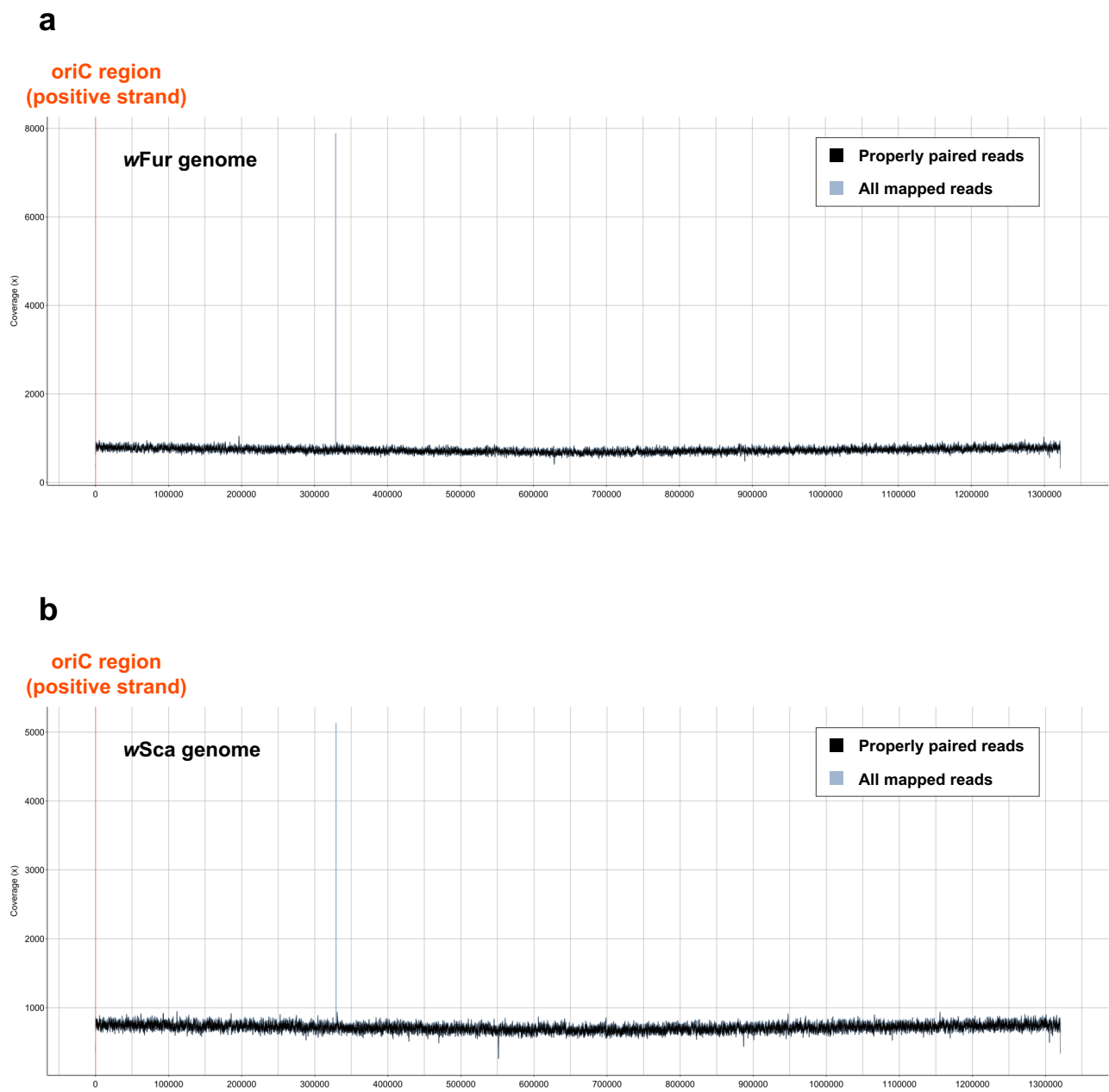

Fig. S5

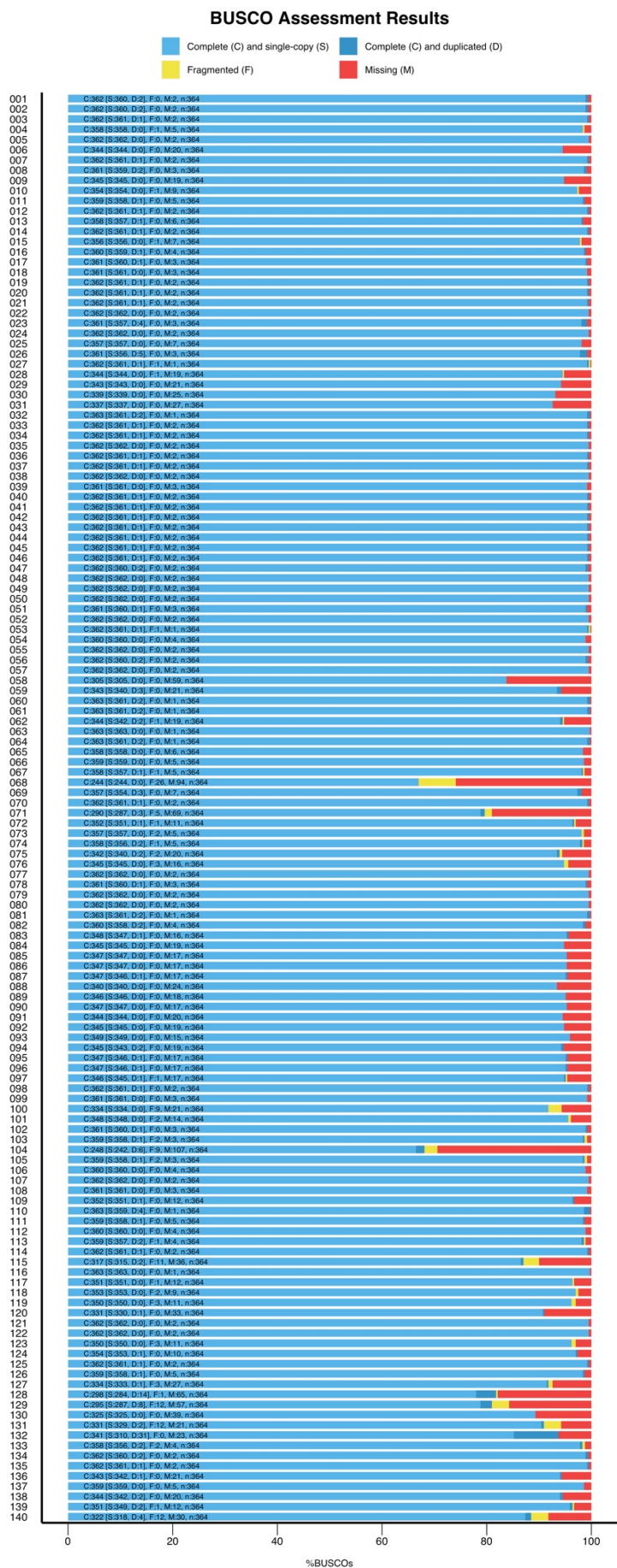

Fig. S6

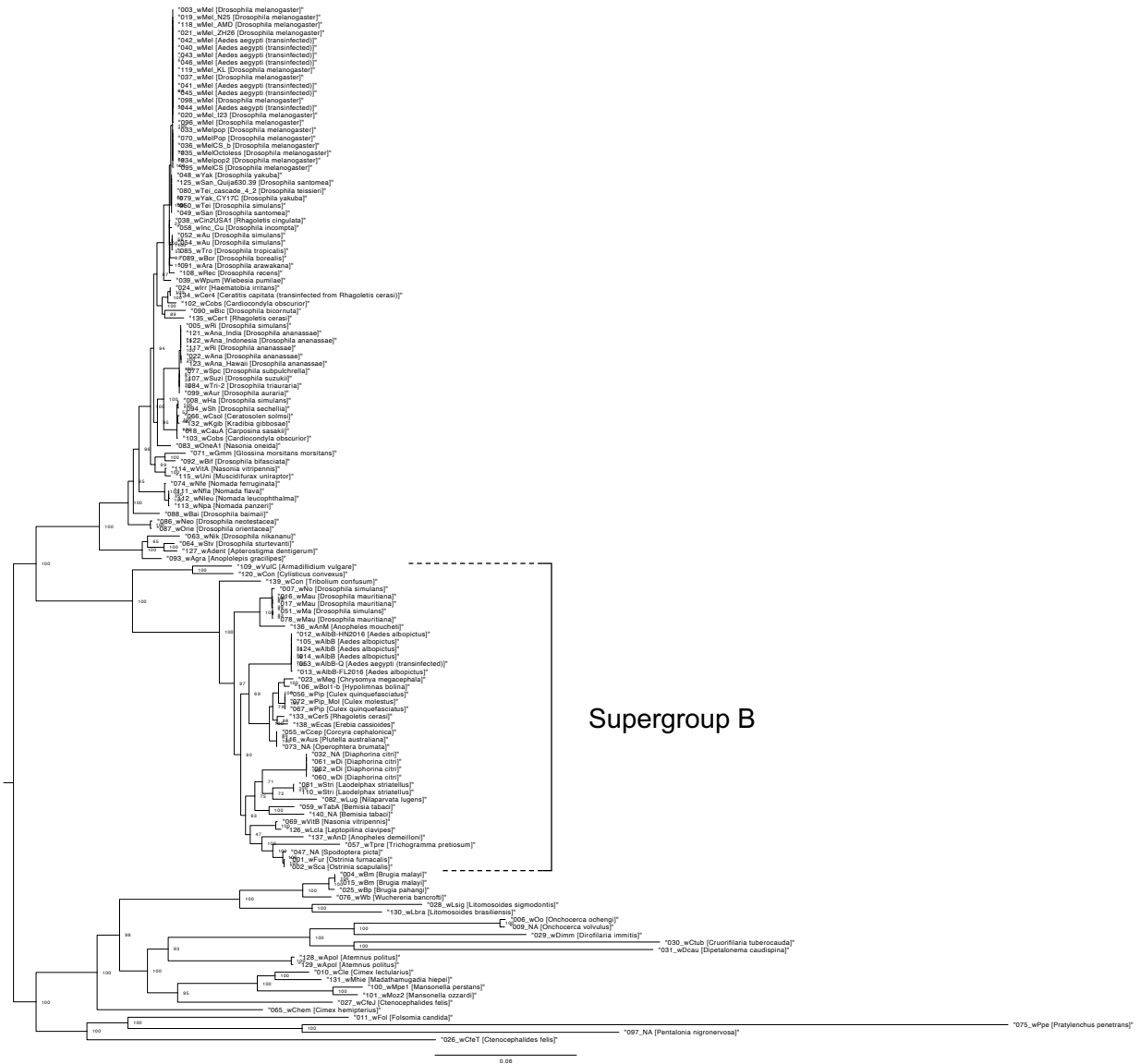

Fig. S7

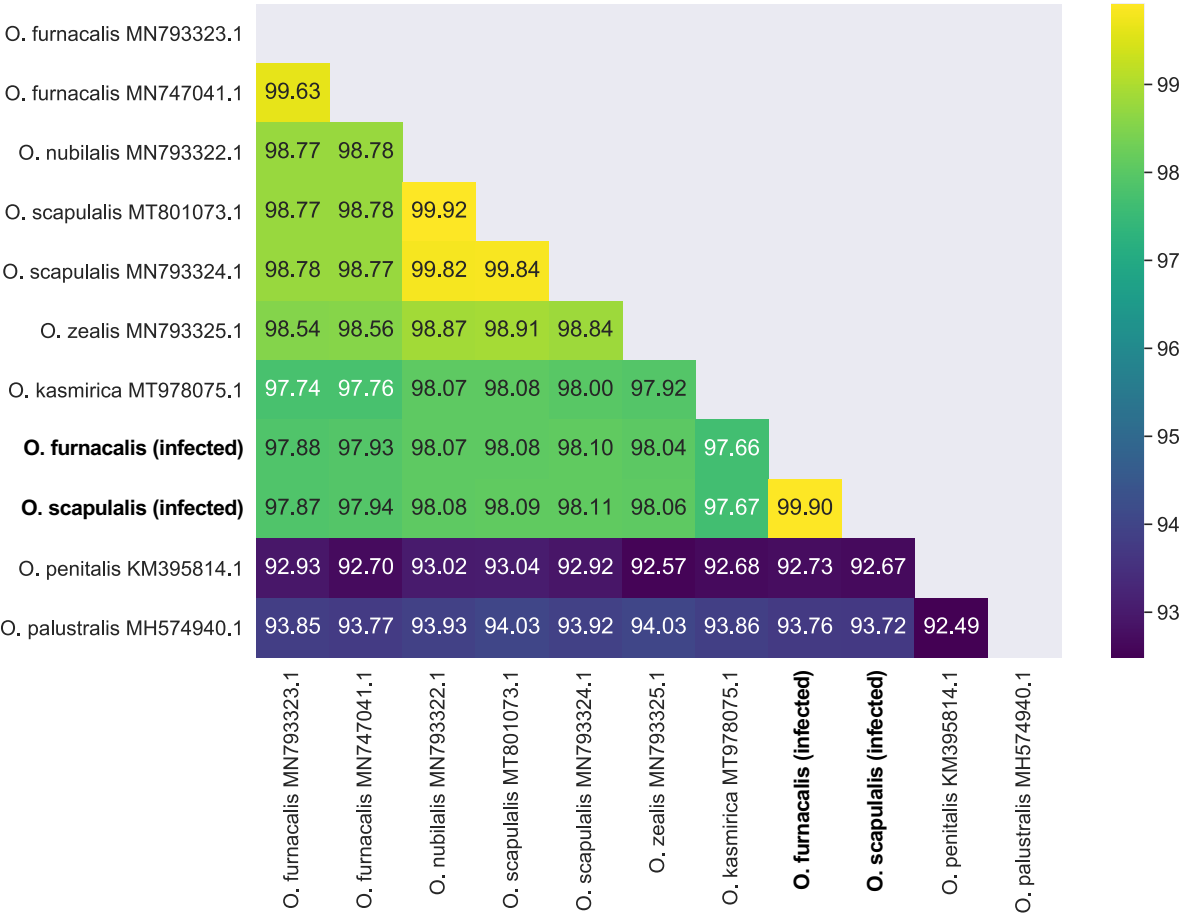

Fig. S8

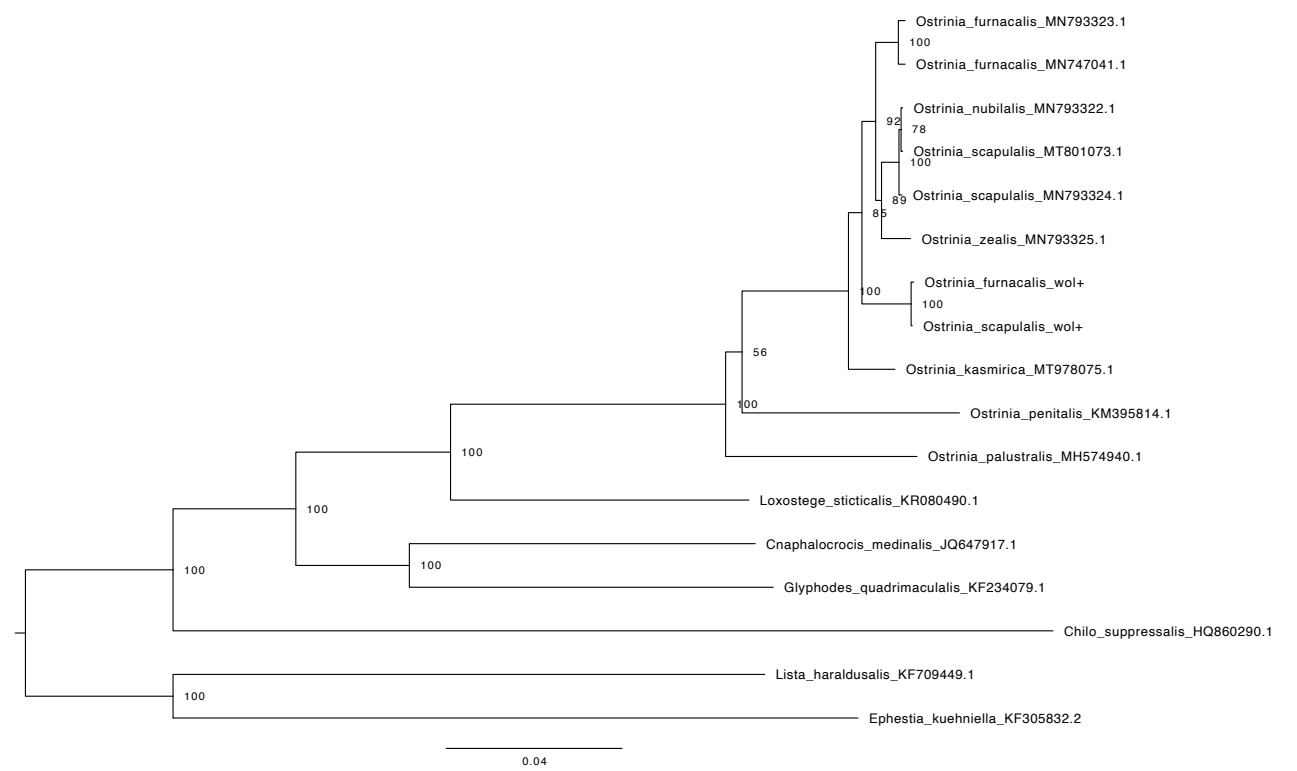

Supplement: Supplementary file 1 — Supplementary file1 (PDF 2948 KB) [file 248_2023_2198_MOESM1_ESM.pdf]
