# Supplementary material for: Two Complete Genomes of Male-Killing Wolbachia Infecting Ostrinia Moth Species Illuminate Their Evolutionary Dynamics and Association with Hosts
Source: Microb Ecol. 2023 Feb 22;86(3):1740–54. doi: 10.1007/s00248-023-02198-7 (PMC10497655; doi:10.1007/s00248-023-02198-7)
Supplement: Supplementary file 2 — Supplementary file2 (DOCX 35 KB) [file 248_2023_2198_MOESM2_ESM.docx]

Supplementary information for:

**Two complete genomes of male-killing *Wolbachia* infecting *Ostrinia* moth species illuminate their evolutionary dynamics and association with hosts**

Tomohiro Muro^1^, Hiroyuki Hikida^1,2^, Takeshi Fujii^3^, Takashi Kiuchi^1^, and Susumu Katsuma^1^*

^1^ Department of Agricultural and Environmental Biology, Graduate School of Agricultural and Life Sciences, The University of Tokyo, Bunkyo-ku, Tokyo, Japan

^2^ Institute for Chemical Research, Kyoto University, Uji, Kyoto, Japan

^3^ Faculty of Agriculture, Setsunan University, Hirakata, Osaka, Japan

*Corresponding author: Susumu Katsuma, Department of Agricultural and Environmental Biology, Graduate School of Agricultural and Life Sciences, University of Tokyo, Yayoi 1-1-1, Bunkyo-ku, Tokyo 113-8657, Japan

Phone: 81-3-5841-8994. Fax: 81-3-5841-8993. E-mail: skatsuma@g.ecc.u-tokyo.ac.jp

**Fig. S1** Systematic schemes of *Ostrinia* moths. The phylogenetic relationships (Kim et al., 1999; Luo et al., 2021; Yang et al., 2021), female sex pheromone components (Ishikawa et al., 1999), and two proposed systematics (Mutuura and Munroe, 1970; Yang et al., 2021) of representative species are summarized. Only clades with high confidence inferred from both genomic and mitochondrial data are shown in the cladogram. Species not distributed in Japan are shown in gray.

**Fig. S2** Coverage distribution of short-reads mapped to (**a**) *w*Fur and (**b**) *w*Sca draft contigs. BWA-MEM program was used to map Illumina reads against the whole Canu assembly with default parameters, and the number of mapped reads was counted at every genome position. The estimated oriC regions are highlighted. Note that the draft contigs have the overlap regions at the beginning and end of the sequence.

**Fig. S3** Coverage distribution of long-read assembly. Dots represent individual contigs in the genome assembly for (**a**) *w*Fur-infected *O. furnacalis* and (**b**) *w*Sca-infected *O. scapulalis* constructed by Canu. Note that a contig corresponding to the mitochondrial genome (shown in blue) is approximately 2.8 times longer than the actual mitochondrial genome due to repetitive assembly of the genome.

**Fig. S4** Coverage distribution of short-reads mapped to (**a**) *w*Fur and (**b**) *w*Sca genome sequences. BWA-MEM program was used to map Illumina reads against the *Wolbachia* genome sequences with default parameters, and the number of mapped reads was counted at every genome position. Both the coverage of all mapped reads and that of properly paired reads are shown. The estimated oriC regions are highlighted.

**Fig. S5** Quality evaluation of *Wolbachia* genome assemblies. A graphical representation of the BUSCO analysis in protein mode for 140 assemblies is shown. Each assembly is labeled with the identification number found in Supplementary Table S1.

**Fig. S6** Phylogenetic relationship of 138 *Wolbachia* genomes. The maximum likelihood tree constructed from concatenated protein sequences of 63 single-copy orthologs is shown. The identification numbers, strain names, and host species are labeled. If no suitable strain name is available, it is denoted by “NA”. Branch support calculated using 1000 replicates of ultrafast bootstrap is shown on the nodes.

**Fig. S7** Graphical representation of ANI values for mitochondrial genomes of *Ostrinia* species. ANI values between 11 mitochondrial genomes of *Ostrinia* moths are calculated by fastANI. The sequence determined in this study are indicated in bold.

**Fig. S8** Phylogenetic relationship of mitochondrial genomes of *Ostrinia* and allied moth species based on nucleotide sequences. The maximum likelihood tree constructed from concatenated nucleotide sequences of 13 protein-coding genes, tRNAs and rRNAs is shown. Two pyralid species (*Lista haraldusalis* and *Ephestia kuehniella*) were used as outgroups. Branch support calculated using 1000 replicates of ultrafast bootstrap is shown on the nodes.
